# Non-redundant roles of the CCR1 and CCR2 chemokine axes in monocyte recruitment during lung metastasis

**DOI:** 10.1016/j.neo.2024.101089

**Published:** 2024-11-19

**Authors:** Alessia G. Liner, Merel van Gogh, Marko Roblek, Matthias Heikenwalder, Lubor Borsig

**Affiliations:** a Institute of Physiology, University of Zurich, Switzerland; b Division of Chronic Inflammation and Cancer, German Cancer Research Center (DKFZ), Heidelberg, Germany; c The M3 Research Center for Malignome, Metabolome and Microbiome, Faculty of Medicine, University of Tuebingen, Otfried-Müller-Straße 37, 72076, Tübingen, Germany; d Comprehensive Cancer Center Zurich

**Keywords:** lung metastasis, monocyte recruitment, macrophages, CCR1, CCR2

## Abstract

Monocytes and monocyte-derived macrophages facilitate cancer progression and metastasis. Inflammatory monocytes expressing CCR2 are actively recruited to metastatic lungs, where they promote tumor cell extravasation, metastatic outgrowth, and an immunosuppressive environment. The role of CCR1 in this process has remained unclear. We used Ccr1- and Ccr2-deficient mice and two different tumor cells lines, MC38 and LLC1 with and without Ccl2-deficiency *in vitro* and *in vivo*. The recruitment of both Ccr1- and Ccr2-deficient monocytes towards the Ccl2 chemokine was significantly impaired, while no substantial recruitment was observed towards Ccl5 *in vitro*. MC38 and LLC1 Ccl2-deficient tumor cells showed reduced lung metastasis in both Ccr1- and Ccr2-deficient mice when compared to wild-type mice. We detected reduced numbers of macrophages and myeloid cells in both chemokine receptor-deficient mice. Lung metastasis in both Ccr1- and Ccr2-deficient mice could be rescued to the same levels as in wild-type mice by an adoptive transfer of Ccr2-deficient but not Ccr1-deficient monocytic cells. Accumulation of Ccr1-deficient monocytes in the lungs was severely impaired upon intravenous monocyte injection, indicating the importance of this axis in cell recruitment. Moreover, the efficient recruitment of adoptive transferred Ccr2-deficient monocytes to the lungs and the restoration of lung metastasis suggests an involvement of an additional, Ccr2-independent chemokine pathway. This data defines the non-redundant functions of the Ccr1- and Ccr2-chemokine axes in monocyte recruitment and macrophage presence during lung metastasis. While Ccr2 is essential for the release of monocytes from the bone marrow, Ccr1 is primarily responsible for monocyte presence at metastatic sites.

## Introduction

The role of chemokines during cancer progression is multifaceted, as illustrated by the widespread expression of chemokines and their receptors in the tumor microenvironment [1,2]. During tumorigenesis, chemokines not only affect tumor cells, endothelial cells, and other stromal cells, but also regulate critical aspects of immune cell biology, such as immune cell recruitment, activation, function, and phenotype. Moreover, there is a significant degree of redundancy, wherein multiple chemokines can bind to various receptors and *vice versa*, making the identification of specific chemokine axes a challenging endeavor.

Elevated levels of chemokines, such as CCL2 and CCL5, in tumors of patients with breast, colon, and prostate cancer correlate with metastatic progressions and poor prognosis [3,4]. CCL2 and CCL5 expression was shown to promote the recruitment of inflammatory monocytes and facilitate the infiltration of pro-tumorigenic macrophages during metastasis [[5], [6], [7]]. For instance, increased CCL5 expression by activated endothelial cells during lung metastasis resulted in monocyte recruitment, while CCR5 antagonist treatment reduced tumor cell survival and inhibited metastasis [5]. Further support for CCL5 involvement in metastasis was obtained in murine breast tumor models, where enhanced CCL5 expression correlated with lung and liver metastases, which were effectively inhibited by blocking CCL5 signaling [8,9]. In addition, CCL5 modulates the recruitment of both regulatory T cells (Tregs) and CD8^+^ T cells [1,10]. On the contrary, elevated CCL2 levels in colon cancer drives the recruitment and activation of myeloid cells, resulting in T cell suppression [11]. In a breast cancer model, tumor and stromal-derived CCL2 induces the recruitment of inflammatory monocytes, while the inhibition of CCL2 signaling blocks this recruitment resulting in attenuation of metastasis [7].

The recruitment and differentiation of inflammatory monocytes into tumor-associated macrophages, which can facilitate metastasis, are mediated by the CCR1 and CCR2 chemokine axes [3,7,[12], [13], [14]]. During lung metastasis, monocyte recruitment is dependent on the CCL2-CCR2 chemokine axis in breast, lung, and colon cancer models [3,7,12,13]. Notably, the CCL2-CCR2 axis is required for the successful egress of inflammatory monocytes from the bone marrow [15,16], which is reflected by the minimal numbers of circulating monocytes in Ccr2-deficient mice [12]. The maintenance and the polarization of macrophages during lung metastasis is dependent on CCR1 expression and macrophage derived CCL3 [6]. Interestingly, inhibiting CCR1-signaling resulted in an expansion of alveolar macrophages and reduced metastatic outgrowth in a melanoma experimental lung metastasis model, whereas blocking CCR2-signaling increased metastasis, which was associated with increased numbers of inflammatory monocytes [17]. On the other hand, the CCR1 chemokine receptor was shown to facilitate liver metastasis by inducing myeloid cell recruitment [18,19], suggesting a tissue-specific difference in chemokine signaling during metastasis. Specifically, tumor derived CCL15 expression resulted in the recruitment of CCR1^+^ cells to the invasive front of colorectal cancer in humans [18].

The expression of chemokines, such as CCL2, CCL3 and CCL5, in the tumor microenvironment in both human and murine models are associated with enhanced recruitment of myeloid-derived cells including macrophages. To dissect the contribution of the CCR1- and CCR2-signaling pathways to monocyte/macrophage recruitment, we used lung and colon tumor models in Ccr1- and Ccr2-deficient mice in combination with adoptive transfer of monocytic cells during the early phase of metastasis. Here we provide evidence for non-redundant functions of both chemokine-signaling pathways during the formation and maintenance of the lung metastatic microenvironment.

## Methods and materials

### Cell culture

Lewis Lung Carcinoma cells, LLC1.1 were obtained from ATCC in 2013; and mouse colon adenocarcinoma cells, MC38 cells were obtained from Dr. J. Schlom (NIH, Bethesda, MD) in 1997. All cell lines were cultured in DMEM high glucose containing L-glutamine (Sigma, D5796), supplemented with 10 % fetal bovine serum (FBS; Biochrom, Germany, S-0615), non-essential amino acids (Thermo Fischer, 11140) and 1mmol/L Na-pyruvate (Thermo Fischer, 11360070), without antibiotics; and kept at 37°C/5 % CO_2_. Cells were expanded into low passage number and working stocks frozen down. All cell lines were tested and found to be free of Mycoplasma, while no further cell authentication assays were carried out. Conditioned medium (CM) from tumor cells was prepared by incubating cells at 50 % confluency in DMEM/2 % heat-inactivated (h.i.) FBS for 24 hours. The CM was centrifuged, filtered (0.45 μm pores) and stored at 4°C.

### Preparation of Ccl2-knock-down tumor cells using lentiviral transduction

Wild-type (wt) LLC1.1 and MC38-GFP cells were transduced with MISSION shRNA Lentiviral Particles (Sigma-Aldrich) containing shRNA targeting CCL2 (NM_011333) and a backbone containing a puromycin resistance gene (pLKO.1-puro). Cells were transduced in the presence of Polybrene (8 μg/ml) and selected using puromycin (3 μg/ml and 20 μg/ml, respectively). Limiting dilution of resistant cells (10 cells/well), LLC1.1-Ccl2KD or MC38-Ccl2KD, were selected and characterized for the chemokine expression and secretion by qPCR and Bio-Plex Multiplex Immunoassay, respectively.

### RNA isolation and quantitative real-time PCR

RNA was extracted from cultured cells using TRI Reagent (Sigma-Aldrich) and isolated using the Direct-zol RNA Miniprep Plus Kit (Zymo Research, R2070). RNA quantity and quality was measured using the NanoDrop 2000 (Thermo Scientific). cDNA was synthesized using the Omniscript RT Kit based on 1 μg of total RNA (Qiagen, 2051511) and qPCR was performed with KAPA SYBR FAST qPCR Master Mix (KAPA Biosystems, KK4602) using specific primers (Supplementary Table S1), 5 μl of a template (1:25 dilution) in duplicates, and analyzed with a CFX96 Touch Real-Time PCR (Bio-Rad) using 2^–ΔΔCT^. Gene expression was normalized against the housekeeping gene Gapdh and relative to the control samples.

### Bio-Plex Multiplex Immunoassay

Chemokine levels in the CM of different tumor cell lines were quantified with the Bio-Plex Pro Mouse Chemokine Panel 31-Plex Assay (Bio-Rad, 12009159) and analyzed using the Bio-Plex 200 System (Bio-Rad) according to company protocol.

### Mice

Wild type C57BL/6J mice (BL6) and Ccr2-deficient (*Ccr2^-/-^*) mice were purchased from Charles River Laboratories and The Jackson Laboratory, respectively. Ccr1-deficient (*Ccr1^-/-^*) mice [20]were kindly provided by Dr. B. Luckow [21]. Mice (male and female, 6–8 weeks old) were randomly assigned to experimental groups, housed in SPF conditions on a 12h light/dark cycle with access to chow and water ad libitum. All animal experiments were approved by the Veterinary Office of Kanton Zurich Switzerland and performed according to the Swiss Animal Protection Law.

For bone-marrow chimeras, recipient mice were irradiated with two doses of 225 kV, 17.7 mA (450 rad) for 180 s using the RS2000 Small Animal Irradiator (Rad Source Technologies) 4h apart. Mice were intravenously (i.v.) injected with ten million bone marrow cells extracted from the femur and tibia of *Ccr2^-/-^* mice 16 h after the second irradiation. Reconstituted mice received Borgal (0.1 %) in the drinking water for the following two weeks.

### Experimental metastatic model

LLC1.1 wt and Ccl2KD tumor cells (300’000) and MC38-GFP wt and Ccl2KD tumor cells (500’000) were i.v. injected into the tail vein of mice. The animals were terminated at day 15 and day 21, respectively, and the lungs were perfused with PBS before collection. The metastatic foci were counted, and the lungs were prepared for histological analysis.

### Spontaneous metastatic model

Mice were subcutaneously (s.c.) injected in the left flank with LLC1.1 wt or Ccl2KD cells (300’000). Tumor size was measured using a digital caliper and the tumor volume was calculated: V=π*L*W2/6. Primary tumors were removed after 14–17 days; and histologically analyzed. Mice were terminated 3 weeks after the tumor removal. The lungs were analyzed by flow cytometry.

### Isolation of CD115^+^ monocytes from the bone marrow

The femur and tibia were flushed with FACS Buffer (PBS/5mM EDTA/2 % FBS) and erythrocytes were lysed with Ammonium-Chloride-Potassium (ACK) lysis buffer. The bone marrow was enriched for monocytes by magnetic-activated cell sorting using a biotinylated anti-mouse CD115 antibody (AFS98, BioLegend) and Streptavidin MicroBeads using MS columns (both Miltenyi Biotec).

### *In vitro* migration assay

Bone marrow (BM)-derived CD115^+^ monocytes (100’000) were seeded into transwell inserts with a polycarbonate membrane with, 5.0 μm pores (Corning) for 4 h at 37°C. DMEM/2 % h.i. FBS with or without recombinant Ccl2 (10 ng/ml), recombinant Ccl5 (10 ng/ml) or a combination of both (20 ng/ml total; from PeproTech) was placed in the lower chamber. Alternatively, CM derived from tumor cell cultures was used as an attractant in the lower chamber. The number of migrated CD115^+^ cells was quantified by flow cytometry after staining with Zombie Fixable Viability Kit (Biolegend) for 30 min on ice. Normalized migration was calculated as the number of migrated monocytes divided by the number of cells migrated in DMEM/2 % FBS h.i. medium without chemokines.

### Flow cytometry analysis

All flow cytometry analysis were acquired on BD FACSCanto II (3L) and analyzed using FlowJo software v. 7.6.5 (TreeStar).

For the analysis of enriched BM-derived monocytes, cells were stained with Zombie Fixable Viability Kit (Biolegend) for 30 min in PBS on ice. Samples were incubated for 10 min with CD16/32 Fc block (Biolegend) and subsequently incubated with antibodies (Supplementary Table S2) in FACS buffer (PBS/10mM EDTA/2 % FBS) for 30 min on ice. After washing with FACS buffer, data was acquired.

Analysis of lung tissue: lungs were perfused with PBS before collection. Metastatic foci were resected from the lung tissue, minced, and digested with Collagenase A and Collagenase D (1 mg/ml each, Sigma-Aldrich) in RPMI/2 % FBS for 30 min at 37°C. The digest was filtered through a 100 μm cell strainer and erythrocytes were lysed on ice with Ammonium-Chloride-Potassium (ACK) lysis buffer. The suspension was filtered through a 40 μm cell strainer and cells were stained with Zombie Fixable Viability Kit (Biolegend), CD16/32 Fc block (Biolegend) and the indicated antibodies (Supplementary Table S2) in FACS buffer on ice. CountBright absolute counting beads (Life Technologies) were used to calculate absolute numbers.

### Adoptive transfer of CD115^+^ monocytes

BM-derived CD115^+^ monocytes were resuspended in Hank's Balanced Salt Solution (HBSS) with 10 μM CXCR4 chemokine receptor antagonist (AMD3100, Tocris). In the experimental metastasis mouse model, CD115^+^ monocytes (1’000’000) were i.v. injected 6 hours after the tumor cell injection and mice were terminated on day 15. In the spontaneous metastatic model, CD115^+^ monocytes (1’000’000) were i.v. injected 1, 3 and 5 days post primary tumor removal and mice were terminated on day 21 post tumor removal. Lungs were embedded in OCT Tissue-Tek® (Sakura, 4583) and analyzed by immunofluorescence microscopy.

### Monocyte recruitment assay *in vivo*

BM-derived CD115^+^ monocytes from BL6 and *Ccr2^-/-^* mice were stained with Vybrant® DiD or DiI Cell-Labeling Solution (5μl/ml, Thermo Fisher) respectively. BL6-DiD and *Ccr2^-/-^* -DiI monocytes were washed with HBSS and resuspended at a 1:1 ratio in the presence of 10 μM CXCR4 antagonist (AMD3100, Tocris). *Ccr2^-/-^* mice were i.v. injected with 300’000 MC38 cells, which was followed by the i.v. injection of labeled BM-derived CD115^+^ monocytes (1’000’000) 6 h later. Mice were terminated 24 h post-tumor cell injection, and the lungs were analyzed by flow cytometry. Alternatively, BL6, *Ccr1^-/-^* or *Ccr2^-/-^* mice were i.v. injected with MC38-GFP tumor cells (500’000) and 12- or 24-hour post-injection the monocyte (Ly6C^hi^ cells) recruitment in lungs was quantified by flow cytometry. Mice without tumor cell injection (naïve mice) were used as control animals.

### Spontaneous recruitment assay *in vivo*

BL6, *Ccr1^-/-^* and *Ccr2^-/-^* mice were injected in the left flank with LLC1.1-GFP tumor cells (300’000) and the primary tumor was removed on day 14. Twenty-four h after tumor removal, BM-derived CD115^+^ monocytes (1’000’000) isolated from *Ccr1^-/-^* and *Ccr2^-/-^* mice were stained with Vybrant® DiD Cell-Labeling Solution (5μl/ml, Thermo Fisher), resuspended with 10 μM CXCR4 chemokine receptor antagonist (AMD3100, Tocris) and i.v. injected. Mice were terminated 24 h later, lungs were embedded in OCT Tissue-Tek® (Sakura, 4583) and analyzed.

### Immunohistochemistry

Formalin-fixed paraffin embedded tissue sections (5 μm) were stained with hematoxylin/eosin (H&E). For cryosections, tissues were fixed in 3 % paraformaldehyde and embedded in OCT Tissue-Tek® (Sakura, 4583). Tissue sections (7 μm) were stained with anti-F4/80 (A3-1, Bio-Rad), anti-CD11b antibody (M1/70, Biolegend) and counterstained with DAPI (Sigma-Aldrich). Histology samples were scanned on a Leica DMi8 Thunder Imager and quantified with the LASX Analysis Software. IHC analysis of the metastatic lesion was normalized to total lung area of the H&E staining. The localization of CD11b^+^ and F4/80^+^ cells in metastatic lesions was quantified.

### Statistical analysis

Data are presented as mean values ± SEM, unless stated otherwise. Statistical analysis using Mann-Whitney U-test was performed using the GraphPad Prism software (version 9.2.0).

## Results

### Ccr1- and Ccr2-dependent migration of monocytes *in vitro* is driven by Ccl2

**Fig. 1:**
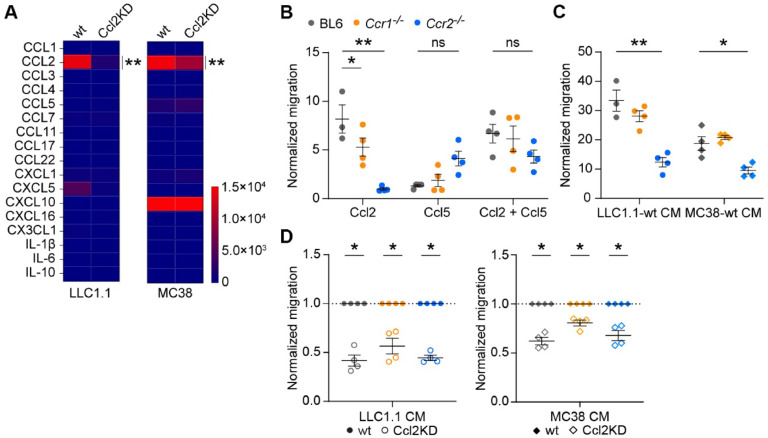
**Tumor cell cytokines induce monocyte migration in Ccr2-and Ccr1-dependent manner. A,** Bio-Plex analysis of chemokines in conditioned medium of LLC1.1 (left) and MC38GFP (right) -wt and -Ccl2KD cells. Data is shown as pg of chemokine per ml of medium. *n* = 6. **B-D,** Migration of BM-derived CD115^+^ monocytes from BL6 (grey), *Ccr1^-/-^* (orange) or *Ccr2^-/-^* (blue) mice towards Ccl2, Ccl5 or both chemokines (**B**); CM of LLC1.1 and MC38-GFP wt cells (**C**) and CM of LLC1.1 wt and Ccl2KD cells and CM of MC38-GFP wt and Ccl2KD cells, respectively (**D**). Data was normalized to the number of migrated monocytes in either DMEM/2 % h.i. FBS (**B-C**) or in the wt CM (**D**). Two independent experiments, *n* = 3-4.

To assess the role of tumor-derived chemokines on metastasis, we prepared Ccl2 knock-down (Ccl2KD) tumor cells using shRNA lentivirus transduction. Ccl2-knock down was confirmed in Lewis Lung Carcinoma LLC1.1 and colon carcinoma MC38-GFP cells by real-time PCR (Supplementary Fig. 1 A). The Bio-Plex analysis of secreted chemokines in the conditioned medium (CM) of LLC1.1-Ccl2KD and MC38-GFP Ccl2KD cells confirmed the downregulation of Ccl2 expression by 90 % and 40 %, respectively (Fig. 1 A). Ccl2KD did not affect tumor cell proliferation (Supplementary Fig. 1 B).

Next, we tested the capacity of the Ccl2 and Ccl5 chemokines to recruit monocytes using a transwell migration assay. We prepared monocytes from the bone marrow of C57BL/6J (BL6), *Ccr1^-/-^*, and *Ccr2^-/-^* mice using CD115 antibody, which contained 50–70 % Ly6C^hi^ monocytes of myeloid cells (Supplementary Fig. 1 C). The migration of *Ccr1^-/-^* monocytes towards the Ccl2 chemokine was reduced when compared to BL6 monocytes, while virtually no migration of *Ccr2^-/-^* monocytes was detected (Fig. 1 B). The Ccl5 chemokine induced a minimal migration of *Ccr2^-/-^* monocytes, while virtually no migration of BL6 or *Ccr1^-/-^* monocytes was observed. Combined Ccl2/Ccl5 chemokines had no additional effects on monocyte migration (Fig. 1 B). The migration of *Ccr2^-/-^* monocytes towards CM derived from both LLC1.1 and MC38-GFP wt cells was reduced when compared to BL6 or *Ccr1^-/-^* monocytes (Fig. 1 C). Of note, both tumor cell lines produce minimal levels of Ccr1-specific chemokines (Fig. 1 A). The migration of monocytes derived from BL6, *Ccr1^-/-^* and *Ccr2^-/-^* mice was dependent on tumor cell-derived Ccl2 in the CM, since migration towards CM from Ccl2KD tumor cells was reduced (Fig. 1 D).

### Ccr1- and Ccr2-dependent monocyte recruitment facilitates lung metastasis

**Fig. 2:**
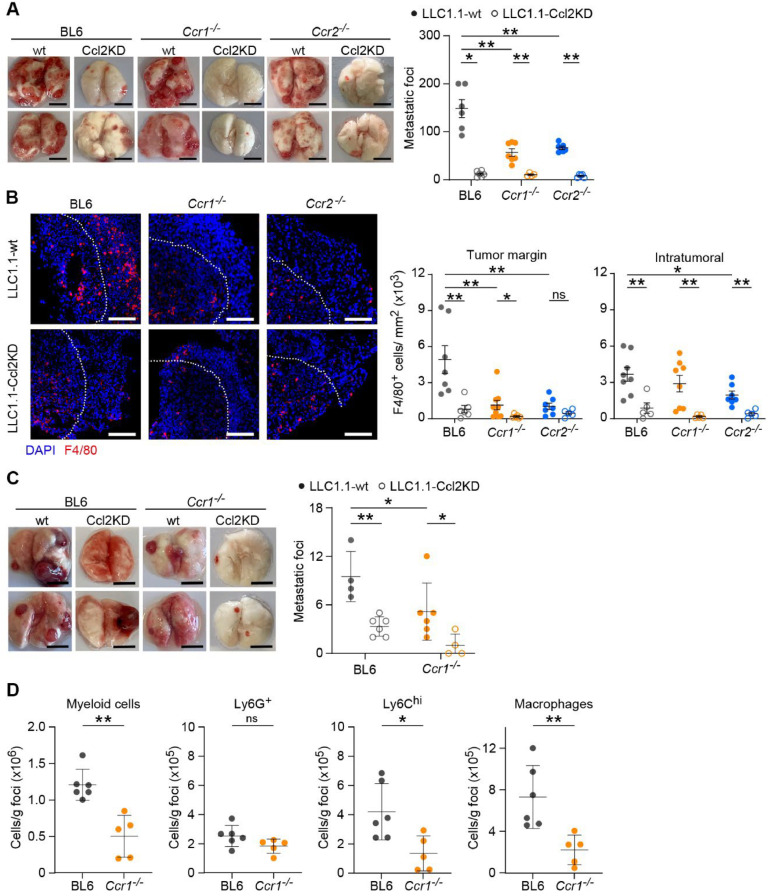
**Ccr1- and Ccr2-dependent monocyte recruitment facilitate lung metastasis. A-B,** LLC1.1 cells (wt or Ccl2KD) were intravenously injected in BL6 (grey), *Ccr1^-/-^* (orange) or Ccr2*^-/-^* (blue) mice and metastasis were assessed on day 15. **A,** Quantification of metastatic foci (right panel) with representative pictures of metastatic lungs (left panel). Scale bar, 5 mm; *n* = 4–7. **B,** Representative pictures of lung metastatic foci (left panel) with F4/80^+^ cells (red) counterstained with DAPI (blue). Dotted line (white) represents the separation of the growing margin in the metastatic foci (towards upper right corner) and the centre of the foci (down left). Quantification of F4/80^+^ cells per mm^2^ in both the margin (middle panel) and inside the metastatic foci (right panel). Scale bar, 100 μm; *n* = 4–7; each tissue was analyzed at 2–3 different tissue depths (dot = tissue section). **C-D,** LLC1.1 cells (wt or Ccl2KD) were subcutaneously injected in BL6 (grey) and *Ccr1^-/-^* (orange) mice, the primary tumor was removed at day 15 and lung metastasis analyzed after an additional 21 days. **C,** Quantification of metastatic foci (right panel) with representative pictures of metastatic lungs (left panel). Scale bar, 1 cm; *n* = 4–7. **D,** Flow cytometry analysis of metastatic foci from BL6 and *Ccr1^-/-^* mice for myeloid cells (CD45^+^CD11b^+^), neutrophils (CD45^+^CD11b^+^Ly6G^+^), Ly6C^hi^ monocytes (CD45^+^CD11b^+^Ly6C^hi^Ly6G^-^) and macrophages (CD45^+^CD11b^+^CD11c^+^F4/80^+^). *n* = 5–6. Data in C-D are presented as mean ±SD. The Mann-Whitney test was used for statistical analysis. ns, not significant; * *P* < 0.05; ** *P* < 0.01.

Mice with a systemic deficiency in Ccr1 and Ccr2 showed reduced metastasis in both lung and liver metastases [6,7,12,19]. To test the effect of individual Ccr1- and Ccr2-deficiences, we tested LLC1.1 tumor cells in an experimental lung metastasis model using BL6, *Ccr1^-/-^*, and *Ccr2*^-/-^ mice. The absence of Ccr1 and Ccr2 resulted in a significant reduction of metastatic outgrowth, compared to the extensive metastasis found in BL6 mice (Fig. 2 A, Supplementary figure 2A). There was no difference in the number of metastasis between *Ccr1^-/-^* and *Ccr2*^-/-^ mice. Tumor cells with reduced Ccl2 production (Ccl2KD) showed attenuated metastatic outgrowth both using LLC1.1 and MC38-GFP tumor models, as shown for two independent clones, irrespective of mouse genotype (Fig. 2 A, Supplementary Fig. 2 B-C).

Next, we assessed the presence of immune cells in the lung metastatic foci. The number of F4/80^+^ macrophages at the growing margin of the metastatic foci was substantially reduced in both *Ccr1^-/-^* and *Ccr2*^-/-^ mice when compared to BL6 mice (Fig. 2 B). In addition, reduced numbers of F4/80^+^ positive cells inside the metastatic lesion were observed in *Ccr2*^-/-^ mice. Similarly, a reduction of CD11b^+^-positive myeloid cells was observed in lung metastatic foci in *Ccr1^-/-^* and *Ccr2*^-/-^ mice, both at the invasive edge and inside the metastatic lesion (Supplementary Fig. 2 D). Metastatic foci from LLC1.1-Ccl2KD tumors revealed even less F4/80^+^ cells in BL6 and *Ccr1^-/-^* mice both at the invasive edge and inside the tumor lesions (Fig. 2 B). There was no significant difference in number of F4/80^+^ cells in *Ccr2^-/-^* mice observed at the growing margin, but only inside the lesions. Similarly, we observed a reduced presence of CD11b^+^ cells in Ccl2KD metastatic foci of BL6 and *Ccr1^-/-^* mice, while no difference in *Ccr2^-/-^* mice was detected (Supplementary Fig. 2 D). Taken together, both Ccr1- and Ccr2-dependent recruitment and stimulation of monocytes/macrophages promote metastasis, which are increased by tumor-derived Ccl2.

### Ccr1-deficiency reduces immune cell infiltration and spontaneous lung metastasis

To validate the relevance of the Ccr1 chemokine axis during cancer progression we used the spontaneous lung metastasis model with subcutaneous injection of LLC1.1 cells. LLC1.1 wt primary tumor growth was comparable in BL6 and *Ccr1^-/-^* mice to LLC1.1-Ccl2KD cells (Supplementary Fig. 2 E-F). We observed a significant reduction of lung metastasis in *Ccr1^-/-^* mice, which was further reduced in mice injected with Ccl2KD tumor cells (Fig. 2 C). The analysis of immune cells in metastatic foci showed a decrease in the number of myeloid cells (CD45^+^CD11b^+^), which was largely due to reduced number of monocytes (CD45^+^CD11b^+^Ly6C^hi^Ly6G^-^), while neutrophils (CD45^+^CD11b^+^Ly6G^+^) remained unchanged in *Ccr1^-/-^* mice when compared to BL6 mice (Fig. 2 D, Supplementary Fig. 2 G). Furthermore, the number of macrophages (CD45^+^CD11b^+^F4/80^+^) was also reduced in *Ccr1^-/-^* mice, indicating a positive correlation between Ccr1-dependent presence of monocytes and macrophages in the metastatic foci and the number of lung metastasis.

### Ccr2 expression on monocytes is dispensable for their recruitment to the metastatic lungs

**Fig. 3:**
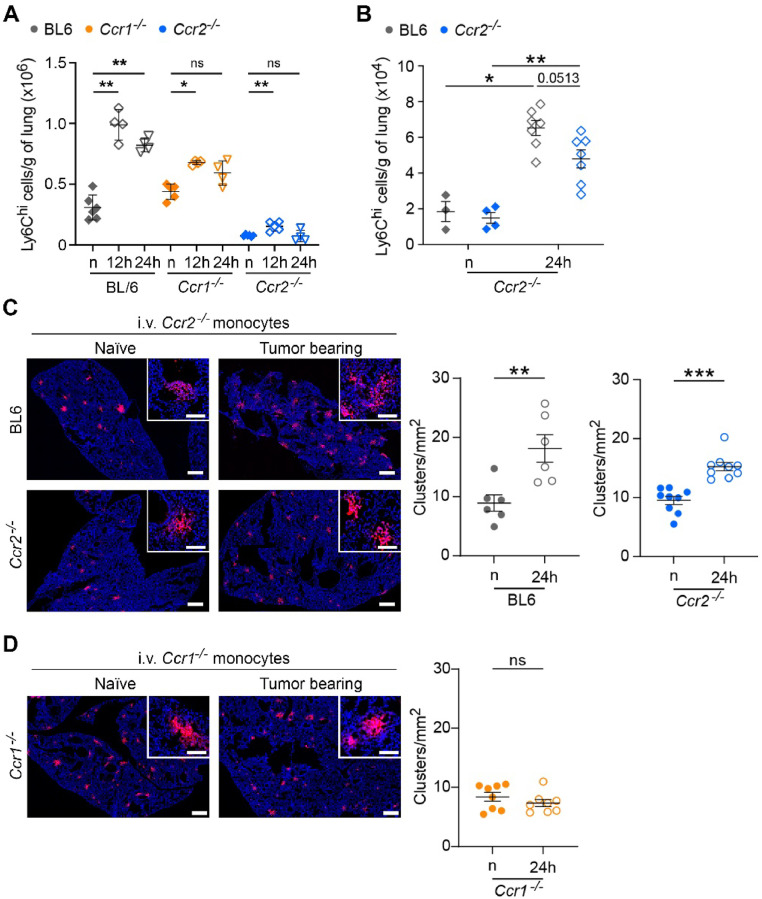
**Circulating Ccr2-deficient monocytes are effectively recruited to early metastatic lungs. A**, BL6, *Ccr1^-/-^,* and *Ccr2^-/-^* mice were i.v. injected with MC38 tumor cells and lungs were analyzed for the recruitment of Ly6C^hi^ monocytes 12- and 24-hour post-tumor cell injection. n = naïve mice without tumor cell injection. Data presented as mean ± SD. *n* = 4–7. **B**, Recruitment of fluorescently labeled CD115^+^ cells isolated from BL6 and C*cr2^-/-^* mice to the lungs of C*cr2^-/-^* mice previously injected with MC38 cells. Monocytic cells were mixed at a 1:1 ratio (BL6:C*cr2^-/-^*). Numbers of recruited monocytes (Ly6C^hi^ cells) to the lungs were analyzed by flow cytometry 24 h post-tumor cell injection. n = naïve mice without tumor cell injection. n=3-7. **C**, Representative images of fluorescently labelled C*cr2^-/-^* CD115^+^ cells (red) in the early metastatic lungs 24 h after their i.v. injection, which was performed one day after LLC1.1 tumor removal. Scale bar, 500 μm; zoom-in, 100 μm. Quantification of CD115^+^ cells recruitment to the lungs of BL6 and C*cr2^-/-^* mice (right panels). Each dot represents a lung section. **D,** Representative images and quantification of *Ccr1^-/-^* CD115^+^ cell recruitment to the early metastatic lungs of C*cr1^-/-^* mice, performed as described in panel C. Each dot represents a lung section. *n* = 3–4 mice. Data in B-D presented as mean ± SEM. The Mann-Whitney test was used for statistical analysis. ns, not significant; * *P* < 0.05; ** *P* < 0.01; *** *P* < 0.001.

Ccl2-mediated monocyte recruitment has previously been shown to be essential for pulmonary metastasis using both Ccl2 depletion or Ccr2-deficient mice [7,12]. However, both approaches affect the Ccl2/Ccr2 axis, which is responsible for the release of monocytic cells from the bone marrow into the circulation [15,16]. To reassess the role of Ccr2-mediated monocyte recruitment to lung metastasis, we analyzed the infiltration of Ly6C^hi^ monocytic cells in the lungs of BL6, *Ccr1^-/-^* and *Ccr2*^-/-^ mice, 12- and 24-h post-intravenous tumor cell injection (Fig. 3 A, Supplementary Fig. 3). The number of Ly6C^hi^ cells significantly increased in the lungs of BL6 (3-fold) and *Ccr1^-/-^* (1.5-fold) mice 12 h after the injection, when compared to naïve mice. Interestingly, a small but statistically significant increase of Ccr2-deficient monocytes was also observed in *Ccr2*^-/-^ mice (2.2-fold), despite the overall reduced numbers of Ly6C^hi^ monocytes in the lungs, indicating that despite the absence of Ccr2 these cells are chemotactically recruited to pre-metastatic lungs. Of note, a significant accumulation of Ly6C^hi^ monocytes was observed only in the BL6 mice 24 h post-tumor cell injection, indicating that both chemokine axes contribute to recruitment and sustained presence of these cells during metastatic initiation. To assess the requirement of Ccr2 for monocyte recruitment during metastatic initiation, we injected fluorescently labeled BL6 and *Ccr2*^-/-^ CD115^+^ enriched monocytes (1:1 ratio) into *Ccr2*^-/-^ mice, which were previously i.v. injected with MC38 cells, and analyzed the lungs 24 h later. The recruitment of both BL6 and *Ccr2*^-/-^monocytes was increased in a similar ratio in tumor-injected mice, when compared to naïve mice (Fig. 3 B).

Next, we assessed the ability of *Ccr1^-/-^* and *Ccr2*^-/-^ monocytes to be recruited to the metastatic lung. BL6, *Ccr1*^-/-^ and *Ccr2*^-/-^ mice were subcutaneously injected with LLC1.1 wt cells, the primary tumor was removed on day 14 and BL6 and *Ccr2^-/-^* mice were i.v. injected with fluorescently labeled *Ccr2^-/-^* enriched CD115^+^ monocytic cells (Fig. 3 C). The quantification of fluorescently labeled cell clusters in the lungs revealed a substantial increase of *Ccr2*^-/-^ monocytes both in tumor bearing BL6 and *Ccr2*^-/-^ mice, when compared to naïve mice (Fig. 3 C). Similarly, *Ccr1*^-/-^ mice were injected with fluorescently labeled *Ccr1*^-/-^ monocytes. However, there was no enhanced recruitment of *Ccr1*^-/-^ monocytes in tumor bearing versus naïve *Ccr1*^-/-^ mice (Fig. 3 D). This data suggests that the absence of Ccr2 on monocytes in the circulation does not hinder their recruitment to early metastatic sites and the active involvement of Ccr1 axis in the recruitment and presence of monocytes at these sites.

### Circulating monocytes determine metastatic initiation

**Fig. 4:**
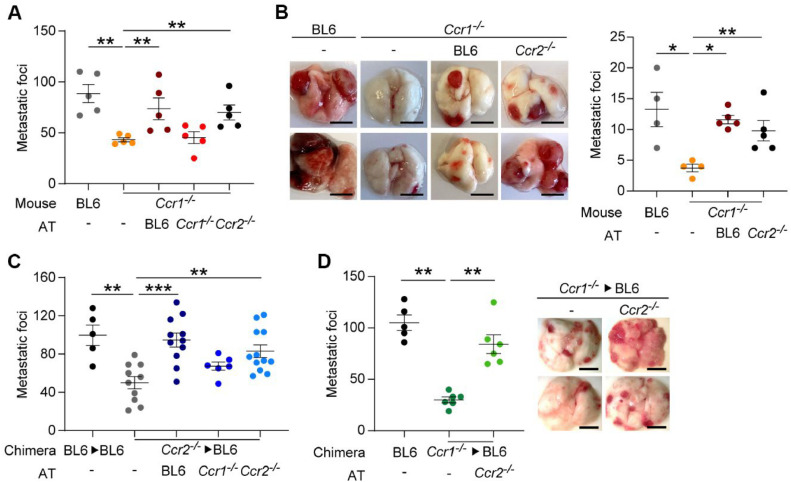
**Intravenous injection of Ccr2-deficient but not Ccr1-deficient monocytic cells restores lung metastasis. A,** Quantification of metastatic foci in an experimental metastasis model after the injection of LLC1.1-wt tumor cells into BL6 or C*cr1^-/-^* mice followed by an adoptive transfer (AT) of BM-derived CD115^+^ monocytic cells isolated from BL6, C*cr1^-/-^*, or C*cr2^-/-^* mice and terminated on day 15. n=5. **B,** Spontaneous lung metastasis of LLC1.1-wt cells. The subcutaneous primary tumor was removed on day 14 and AT of CD115^+^ cells was i.v. injected at 1, 3 and 5 days after tumor removal. Mice were terminated 21 days post-tumor removal. Quantification of metastatic foci (right panel) with representative pictures of metastatic lungs (left panel). Scale bar, 5 mm; *n* = 4–5. **C,** Quantification of metastatic foci in an experimental metastasis model of LLC1.1-wt tumor cells injected in chimeric C*cr2^-/-^*→BL6 mice, followed by an AT with CD115^+^ cells isolated from BL6, C*cr1^-/-^* or C*cr2^-/-^* mice was performed. BL6→BL6 mice were used as a control. *n* = 5–12. **D,** Quantification of metastatic foci in an experimental lung metastasis model of LLC1.1 wt cells injected in chimeric mice (*Ccr1^-/-^*→BL6) followed by AT of CD115^+^ cells isolated from BL6 or *Ccr2^-/-^* mice (left panel) with representative pictures (right panel). n=5-6.

To evaluate the contribution of circulating monocytic cells to metastatic initiation in *Ccr1^-/-^* mice, we used adoptive transfer (AT) of bone marrow-derived CD115^+^ cells, which were injected 6 h after the intravenous injection of LLC1.1 wt cells. AT of CD115^+^ cells derived from BL6 and *Ccr2^-/-^* mice resulted in restoration of lung metastasis in *Ccr1^-/-^* mice (Fig. 4 A, Supplementary Fig. 4A). However, AT of Ccr1-deficient cells had no effect on metastasis, indicating that Ccr1-expression is required for promotion of lung metastasis.

Next, we tested whether the temporal increase in circulating BL6 and *Ccr2^-/-^* monocytes affected spontaneous lung metastasis in *Ccr1^-/-^* mice. One, three and five days after the removal of the primary tumor, ATs with CD115^+^ cells from both BL6 and *Ccr2^-/-^* mice restored metastasis in *Ccr1^-/-^* mice to the same levels as observed in BL6 mice (Fig. 4 B). These results indicate that Ccr2-deficient CD115^+^ cells also promote lung metastasis once these cells are in circulation. Of note, the CD115^+^ cell preparation used in AT is enriched in monocytes (Supplementary Fig. 1C), yet we cannot exclude the contribution of other myeloid-derived cells from these preparations on metastasis.

The individual contributions of the Ccr1 and Ccr2 chemokine axes to modulation of metastasis was assessed also in *Ccr2^-/-^* mice. Since endothelial Ccr2 expression also promotes lung metastasis [12,22], we prepared chimeric BL6 mice and reconstituted them with Ccr2-deficient bone marrow (C*cr2^-/-^*→BL6). C*cr2^-/-^*→BL6 mice showed reduced experimental lung metastasis when compared to control BL6→BL6 mice (Fig. 4 C, Supplementary Fig. 4 B). Notably, AT with Ccr2-deficient but not Ccr1-deficient CD115^+^ cells restored lung metastasis to the same levels as BL6-derived cells. For completion, we prepared C*cr1^-/-^*→BL6 mice, and performed AT using Ccr2-deficient CD115^+^ cells (Fig. 4 D). The reduced lung metastasis observed in chimeric mice could be restored by AT to the levels observed in BL6. These findings confirm that the presence of Ccr2-deficient monocytic cells in the circulation promotes metastasis, while the injection of Ccr1-deficient monocytes had no effect.

## Discussion

Tumor-induced myelopoiesis results in increased numbers of mature and immature myeloid cells in circulation, as well as in the primary and metastatic tumors [23,24]. In this study, focusing on the recruitment of monocytes during metastasis, we demonstrate that Ccr1- and Ccr2-deficiency attenuates lung metastasis to the same extent. Interestingly, this phenotype can be rescued by an adoptive transfer of Ccr2-deficient but not Ccr1-deficient monocytes. We show that Ccr2-deficient monocytes are efficiently recruited to the metastatic sites when present in the circulation. Lastly, we show that Ccl2-knock-down tumor cells in *Ccr1^-/-^* mice leads to virtual impairment of lung metastasis.

Tumor- and stromal-derived chemokines promote the recruitment and the activation of immune cells during metastasis, thereby influencing the metastatic process [1,2,4]. In mammary tumors, Ccl2, Ccl3 and Ccl5 were shown to modulate the infiltration of monocytes, resulting in the accumulation and activation of tumor-associated macrophages, TAMs [24]. Specifically, tumor- and stromal-derived Ccl2 is known to promote monocyte recruitment, which is required for lung metastasis [7]. Similarly, mammary tumor cells with reduced Ccl5 expression attenuated lung metastasis whereas Ccl5 overexpression increased TAMs and rescued metastasis [8,9]. Ccr1 expression was also shown to be essential for the recruitment of immature myeloid cells during liver metastasis in a colon cancer model [19]. In this study, we compared the contribution of the Ccr2 and Ccr1 chemokine receptors in relation to lung metastasis. Monocytes were recruited in a Ccl2/Ccr2 dependent manner. Accordingly, tumor cell-derived supernatant induced monocyte recruitment of wt and *Ccr1^-/-^* monocytes, but not of *Ccr2^-/-^* monocytes, which is in agreement with previous studies [7,13]. Interestingly, we also observed reduced migration of *Ccr2^-/-^* monocytes towards tumor cell-derived supernatant from Ccl2KD cells, indicating an additional Ccl2-dependent mechanism.

Chemokines, including Ccl2, can also promote the presence and activation of macrophages at the metastatic sites [1,2], facilitating tumor cell extravasation and metastasis [7,12]. In our model, we observed reduced numbers of macrophages in metastatic lungs of both *Ccr1^-/-^* and *Ccr2^-/-^* mice, which correlated with attenuated metastasis. The number of circulating monocytes in *Ccr1^-/-^* mice were comparable to BL6 mice [20,21], thus the reduced presence of monocyte/macrophages in metastatic sites is likely due to reduced recruitment. The reduced macrophage population observed in *Ccr2^-/-^* mice is likely due to the minimal numbers of systemic monocytes [12,15]. The significance of *Ccr2^-/-^* monocyte recruitment was shown in the adoptive transfer experiment, wherein these monocytes were efficiently recruited to the early metastatic lung, ultimately resulting in increased metastasis. These data provide evidence for a Ccl2/Ccr2 independent monocyte recruitment during lung metastasis. Contrary to *Ccr2^-/-^* monocytes, adoptive transfer experiments with *Ccr1^-/-^* monocytes did not affect monocyte recruitment nor lung metastasis. While the Ccr1-dependent macrophage retention during metastasis has previously been described [24], our data suggest that Ccr1-deficiency also impairs monocyte recruitment during metastatic initiation. Taken together, these data show that *Ccr2^-/-^* monocytes, once in circulation, can be efficiently recruited to the lung metastatic sites and that Ccr1 facilitates inflammatory monocyte recruitment to pulmonary metastatic sites.

The cytokine analysis of LLC and MC38 tumor cell lines revealed substantial expression of CCL2 and CXCL10, as observed previously [25]. Reduced metastasis with both MC38-Ccl2KD and LLC1.1-Ccl2KD tumor cells correlated with a reduction in macrophages (F4/80^+^ cells) and myeloid cells in the metastatic lesions of BL6 as well as *Ccr1^-/-^* mice. Although we cannot exclude the involvement of chemokines originating from the tumor microenvironment, the strong reduction in metastasis observed with the Ccl2KD tumor cells suggests the critical contribution to this process. While the absence of Ccr1 or Ccr2 individually caused a reduction of macrophages in metastatic lesions, Ccl2KD cells showed reduced macrophages only in *Ccr1^-/-^* mice. The number of macrophages in BL6 mice injected with LLC1.1-Ccl2KD tumor cells was comparable to *Ccr1^-/-^* or *Ccr2^-/-^* mice injected with LLC1.1-wt, suggesting that both chemokine axes act in a subsequent manner.

Besides their chemoattractant function, chemokines also affect other immune cells within the tumor microenvironment, thereby modulating cancer progression [11,26,27]. Ccl2 is known to promote M2-polarization of macrophages [28], which in turn promotes tumor angiogenesis [29,30]. In addition, Ccl2 affects the recruitment of the Treg subpopulation [31]. The CCL5-CCR3 signaling axis induced Th2 polarization of CD4^+^ T cells in a breast tumor model [32]. In colorectal cancer, Ccl2 promotes immunosuppression of T cells by regulating the priming of myeloid-derived suppressor cells [11]. However, in these models the effect of these chemokines on monocyte or macrophages recruitment has not been assessed. We cannot exclude the effect of Ccr1- or Ccr2-deficiency on involvement of other immune cells, yet the short-term rescue of lung metastasis upon adoptive transfer of enriched monocytic cells argues against a chemokine-related immune responses observed in the models described above.

The role of inflammatory chemokine receptors, Ccr1, Ccr2, Ccr3, and Ccr5 during inflammation has been recently studied with different genetically modified mouse models [16,33]. While Ccr2-deficiency significantly reduced the recruitment of monocytic cells to inflammation sites, only the absence of all inflammatory chemokines resulted in a complete absence of their recruitment [16]. Single cell RNA sequencing analysis of murine bone marrow-derived monocytes revealed the majority of monocytes to express exclusively Ccr2, while only a minority of these cells co-express Ccr1 [33,34]. Tissue-resident macrophages downregulate Ccr2 expression and induce Ccr1 expression either alone or in combination with Ccr5. These data indicate a non-redundant and a context dependent expression of chemokine receptors on monocytes and macrophages that are dynamically regulated depending on the tissue microenvironment during homeostasis and inflammation [16,33].

A previous study showed that reduced numbers of Ly6C^hi^ cells in the circulation and in the naïve lungs was similarly reduced both in the Ccr1- and Ccr2-deficient mice [16]. We also observed a reduction in Ly6C^hi^ cell recruitment during lung metastasis in both *Ccr1^-/-^* and *Ccr2^-/-^* mice, which resulted in attenuation of lung metastasis. While the adoptive transfer of the monocyte-enriched population from *Ccr2^-/-^* mice could rescue metastasis in *Ccr1^-/-^* mice, the transfer of such cells derived from *Ccr1^-/-^* mice did not rescue metastasis in *Ccr2^-/-^* mice. Although we cannot exclude the contribution of other myeloid cells co-enriched in the monocytes used for adoptive transfer, the presence of Ccr2^+^ monocytes in the *Ccr1^-/-^* derived-cells did not rescue metastasis, strongly indicating the involvement of Ccr1-dependent axis in this process. A recent study on tumor-associated macrophages in pancreatic cancer models has provided the initial evidence about the heterogeneity of these cells and their development from the monocytes [35]. Further studies focusing on the specific monocyte populations and their chemokine receptor expression will be required to delineate its contribution not only to monocyte recruitment but also macrophage differentiation during metastasis.

## Abbreviations

AT, adoptive transfer; BL6, C57BL/6J mice; BM, bone marrow; CM, conditioned medium; i.v.; intravenous injection; KD; knock-down; LLC; Lewis Lung carcinoma; s.c., subcutaneous injection; TAM, tumor-associated macrophages; Treg, regulatory T cells; wt, wild type.

## Funding

This work was funded by Swiss National Science Foundation grant #310030_152901 and 310030-173076 to LB and ERC Consolidator Grant (HepatoMetaboPath) to MH.

### Data availability statement

Data presented in this study are available from the corresponding author upon a reasonable request.

## CRediT authorship contribution statement

**Alessia G. Liner:** Data curation, Formal analysis, Methodology, Writing – review & editing. **Merel van Gogh:** Data curation, Formal analysis, Methodology, Writing – review & editing, Validation. **Marko Roblek:** Conceptualization, Data curation, Formal analysis, Methodology, Validation, Writing – review & editing. **Matthias Heikenwalder:** Conceptualization, Funding acquisition, Resources, Writing – review & editing. **Lubor Borsig:** Conceptualization, Funding acquisition, Investigation, Resources, Writing – original draft, Writing – review & editing, Validation, Supervision.

## Declaration of competing interest

The authors declare that they have no known competing financial interests or personal relationships that could have appeared to influence the work reported in this paper.

## Appendix Supplementary materials

Image, application 1
