# Supplementary material for: Non-redundant roles of the CCR1 and CCR2 chemokine axes in monocyte recruitment during lung metastasis
Source: Neoplasia. 2024 Nov 19;59:101089. doi: 10.1016/j.neo.2024.101089 (PMC11617888; doi:10.1016/j.neo.2024.101089)
Supplement: Supplementary file 1 [file mmc1.pdf]

Supplementary Data

Liner et al.

Non-redundant roles of the CCR1 and CCR2 chemokine axes in monocyte recruitment during lung metastasis

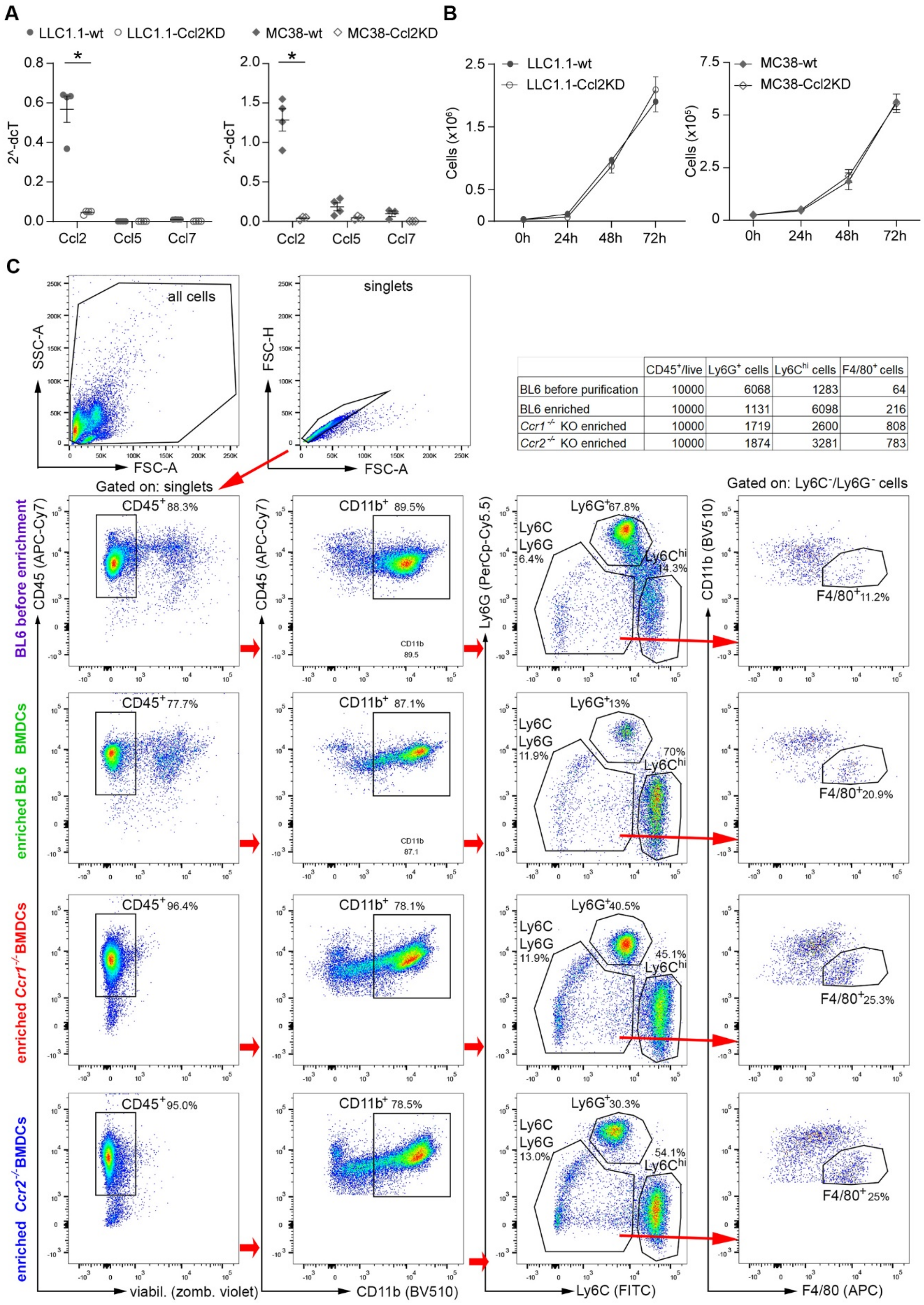

	CD45$^+$/live	Ly6G$^+$ cells	Ly6C^{hi} cells	F4/80$^+$ cells
BL6 before purification	10000	6068	1283	64
BL6 enriched	10000	1131	6098	216
Ccr1$^{-/-}$ KO enriched	10000	1719	2600	808
Ccr2$^{-/-}$ KO enriched	10000	1874	3281	783

Supplementary Figure 1. Ccl2-knock down tumor cells inhibits recruitment of monocytes in Ccr1- and Ccr2-dependent manner. A, qPCR analysis of chemokines from LLC1.1 (left) and from MC38GFP (right) wt and Ccl2KD tumor cells. Three independent experiments, n=3-4**. B,** Cell proliferation assay of LLC1.1 (upper) and MC38GFP (lower) -wt and -Ccl2KD cells *in vitro*. **C,**

Enrichment of CD115^{+} monocytes from the bone marrow using anti-CD115^{+}-antibody based magnetic cell sorting. WT samples before and after enrichment are presented and enriched samples of *Ccr1*$^{-/-}$ and *Ccr2*$^{-/-}$ are shown with percentual populations and absolute number of cells (upper right). The Mann-Whitney test was used for statistical analysis. ns, not significant; *, $P< 0.05$; **, $P< 0.01$; ***, $P< 0.001$.

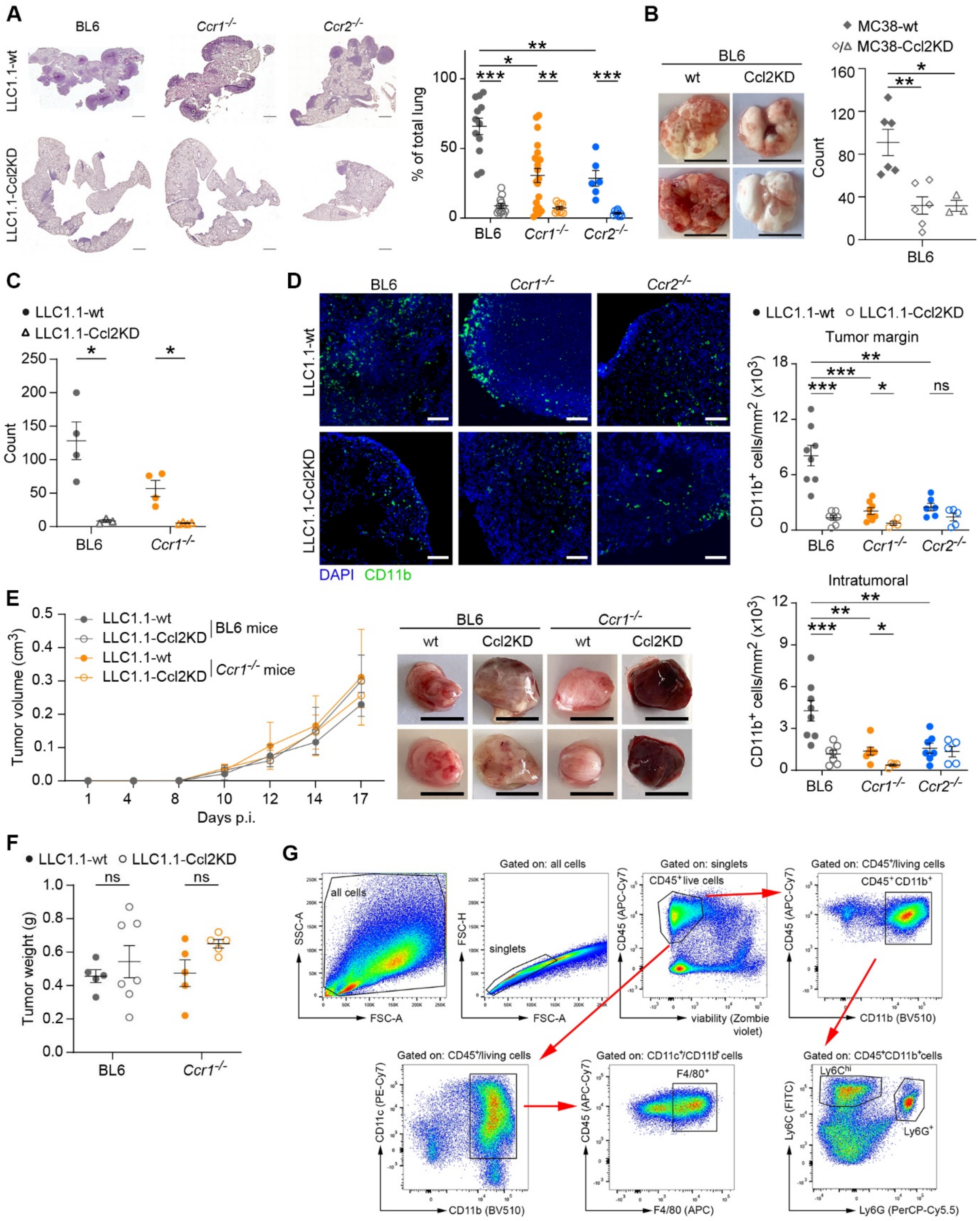

Supplementary Figure 2. Tumor cell derived Ccl2 facilitates growth of experimental lung metastases with both MC38GFP and LLC1.1 tumor cells, while *Ccr1* deficiency does not affect subcutaneous LLC1.1 wt tumor growth. A-D, LLC1.1 cells (wt or Ccl2KD) were intravenously injected in BL6 (grey), Ccr1-/- (orange) or Ccr2-/- (blue) mice and metastasis were assessed on day 15. **A,** Representative images (left panel) of lung sections stained with H&E and metastatic area quantification in the lungs (right panel). Scale bar, 1 mm; n=4-7; each tissue was analyzed at 2-4 different tissue depths (dot = tissue section). **B,** Experimental lung metastasis of MC38GFP-wt and -Ccl2KD tumor

cells in BL6 wt mice on day 21. Representative images of lungs (left panel), and quantification of metastatic foci (right panel) where two different Ccl2KD clones were analyzed (empty circles and triangles). Scale bar, 1 cm. **C,** Quantification of lung metastatic foci of LLC1.1-wt and the second Ccl2KD clone of LLC1.1 (open symbols) in BL6 and *Ccr1*$^{-/-}$ mice on day 15. **D,** Representative pictures of lung metastatic foci (left panel) of CD11b^{+} cells (green) counterstained with DAPI (blue) from BL6, *Ccr1*$^{-/-}$ and *Ccr2*$^{-/-}$ mice i.v. injected with LLC1.1-wt or -Ccl2KD cells. The quantification of CD11b^{+} cells per mm^2 in both the tumor margin (upper panel) and inside the metastatic foci (lower panel). Scale bar, 100 µm; n=4-7; each tissue was analyzed at 2-3 different tissue depths (dot = tissue section). **E-F,** LLC1.1 cells (wt or Ccl2KD) were subcutaneously injected in BL6 and *Ccr1*$^{-/-}$ mice, the primary tumor was removed on day 15 and lung metastases were analyzed after an additional 21 days. **E,** Primary tumor growth in BL6 and *Ccr1*$^{-/-}$ mice (LLC1.1wt and Ccl2KD cells). n=5-7. Representative pictures of primary tumors (right panel); scale bar, 1 cm. **F,** final tumor weight n=5-7. **G,** Flow cytometry gating strategy for the analysis of immune cells from lung metastatic foci. The Mann-Whitney test was used for statistical analysis. ns, not significant; * P< 0.05.

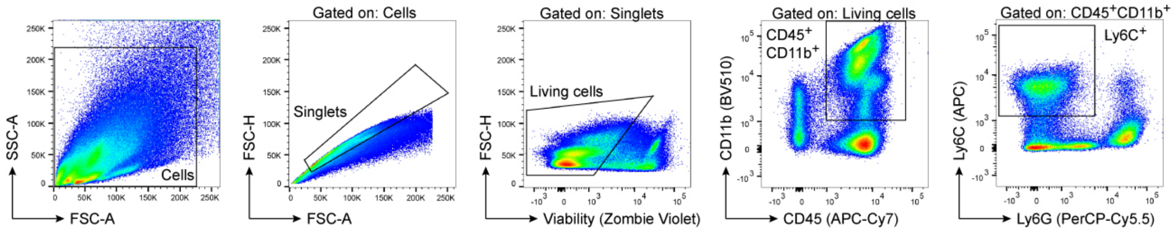

Supplementary Figure 3. Quantification of Ly6C^{hi} monocyte recruitment to the lungs. Gating strategy to analyze Ly6C^{hi} cells recruited to naïve and metastatic lungs using flow cytometry.

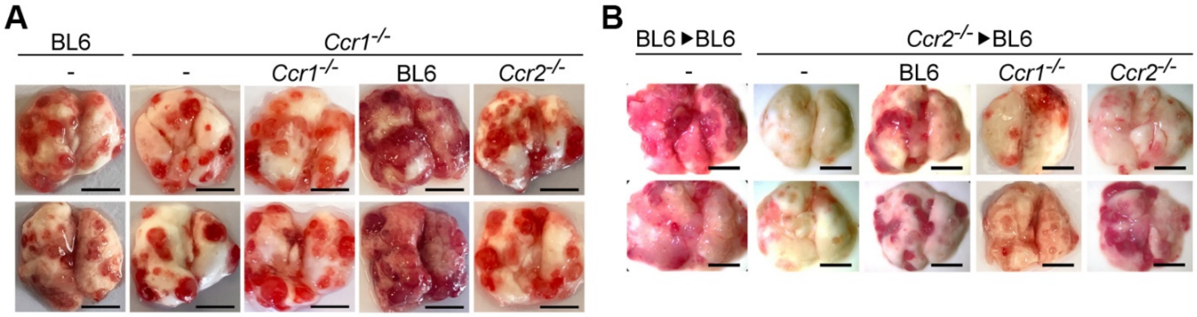

Supplementary Figure 4. Circulating monocytes promote experimental lung metastasis in a Ccr2-independent manner. A, Representative pictures of metastatic lungs from BL6 and *Ccr1*$^{-/-}$ mice i.v. injected with LLC1.1-wt cells followed by AT of CD115^{+} cells isolated from BL6, *Ccr1*$^{-/-}$ or *Ccr2*$^{-/-}$. Scale bar, 1 cm. **B,** Representative pictures of lungs from chimeric mice (BL6→BL6 and C*cr2*$^{-/-}$→BL6) injected with LLC1.1-wt tumor cells. Scale bar, 1 cm.

Supplementary Table 1

qRT-PCR primer sequences

Gene	Forward	Reverse
Ccl2	5’-TTAACGCCCCACTCACCTGC-3’	5’-TGGGGTCAGCACAGACCTCTC-3’
Ccl5	5`-GCTGCTTTGCCTACCTCTCC-3`	5`-TCGAGTGACAAACACGACTGC-3`
Ccl7	5’-TGGGAAGCTGTTATCTTCAAGACA-3’	5'-CTCGACCCACTTCTGATGGG -3'
Ccr1	5’-AAGATCCTCAAAGGCCCAGAAACA-3’	5’-AGTTGTGGGGTAGGCTTCTGTGA-3’
Ccr2	5’-GCAAGTTCAGCTGCCTGCAAA-3’	5’-GTATGCCGTGGATGAACTGAGGT-3’
Ccr3	5’-TCACCAGAGACAAGTAGAATGGCA-3’	5’-AGTGGAGGCAGGAGCCATGA-3’
Gapdh	5’-CATGTTCCAGTATGACTCCAC-3’	5’-GGCCTCACCCCATTTGATGT-3’

Supplementary Table 2

Flow cytometry antibodies

Antigen	Fluorophore	Clone	Company
CD45	APC-Cy7	30-F11	Biolegend
CD11b	BV510/Amcyan	M1/70	Biolegend
CD11c	PE-cy7	N418	Biolegend
F480	APC	A3-1	Bio-Rad
Ly6C	FITC / APC*	HK1.4	Biolegend
Ly6G	PerCP-cy5.5	1A8	Biolegend

*Used for Ly6C^{hi} cell recruitment
